# Prioritized Expression of *BDH2* under Bulk Translational Repression and Its Contribution to Tolerance to Severe Vanillin Stress in *Saccharomyces cerevisiae*

**DOI:** 10.3389/fmicb.2016.01059

**Published:** 2016-07-06

**Authors:** Yoko Ishida, Trinh T. M. Nguyen, Sakihito Kitajima, Shingo Izawa

**Affiliations:** Department of Applied Biology, Graduate School of Science and Technology, Kyoto Institute of TechnologyKyoto, Japan

**Keywords:** *BDH1*, *BDH2*, *ADH7*, vanillin, furfural, translation repression, lignocellulosic biomass, bioethanol production

## Abstract

Vanillin is a potent fermentation inhibitor derived from the lignocellulosic biomass in biofuel production, and high concentrations of vanillin result in the pronounced repression of bulk translation in *Saccharomyces cerevisiae*. Studies on genes that are efficiently translated even in the presence of high concentrations of vanillin will be useful for improving yeast vanillin tolerance and fermentation efficiency. The *BDH1* and *BDH2* genes encode putative medium-chain alcohol dehydrogenase/reductases and their amino acid sequences are very similar to each other. Although *BDH2* was previously suggested to be involved in vanillin tolerance, it has yet to be clarified whether Bdh1/Bdh2 actually contribute to vanillin tolerance and reductions in vanillin. Therefore, we herein investigated the effects of Bdh1 and Bdh2 on vanillin tolerance. *bdh2*Δ cells exhibited hypersensitivity to vanillin and slower reductions in vanillin than wild-type cells and *bdh1*Δ cells. Additionally, the overexpression of the *BDH2* gene improved yeast tolerance to vanillin more efficiently than that of *BDH1.* Only *BDH2* mRNA was efficiently translated under severe vanillin stress, however, both *BDH* genes were transcriptionally up-regulated. These results reveal the importance of Bdh2 in vanillin detoxification and confirm the preferential translation of the *BDH2* gene in the presence of high concentrations of vanillin. The *BDH2* promoter also enabled the expression of non-native genes under severe vanillin stress and furfural stress, suggesting its availability to improve of the efficiency of bioethanol production through modifications in gene expression in the presence of fermentation inhibitors.

## Introduction

Biomass conversion inhibitors including vanillin, furans, and organic acids are inevitably formed during the saccharification pretreatment of the lignocellulosic biomass. They are significant barriers to decreasing the production cost of bioethanol because of their toxicity to yeast cells (Palmqvist and Hahn-Hägerdal, 2000; Helle et al., 2003; Klinke et al., 2004; Jonsson et al., 2013). Because vanillin is one of the most potent fermentation inhibitors in lignocellulose hydrolysates (Klinke et al., 2004), breeding robust yeast strains with a high tolerance to vanillin has become increasingly important for improving the productive efficiency of bioethanol from the lignocellulosic biomass. In order to improve yeast tolerance against vanillin, its physiological effects on yeast cells and cellular responses to vanillin need to be clarified in more detail.

Previous studies reported that several types of stress such as glucose starvation and ethanol stress cause a rapid reduction in overall protein synthesis and the formation of cytosolic mRNP granules including P-bodies and stress granules (SGs) in *Saccharomyces cerevisiae* (Ashe et al., 2000; Kato et al., 2011). We also showed that severe vanillin stress caused translational repression and the formation of cytosolic mRNP granules, leading to a reduction in overall protein synthesis levels and the limited translation of mRNAs (Iwaki et al., 2013b; Nguyen et al., 2014). It is conceivable that mRNAs encoding the proteins that play a role in stress tolerance are efficiently translated even under severe stress conditions. Indeed, small heat shock protein mRNAs are efficiently translated overcoming translation repression caused by glucose starvation (Zid and O’Shea, 2014). We also recently reported that *ADH7* mRNA, which encodes a NADPH-dependent medium-chain alcohol dehydrogenase, was efficiently translated under severe vanillin stress conditions (Larroy et al., 2002; Nguyen et al., 2015). Adh7 and Adh6 reduce vanillin to vanillyl alcohol (Larroy et al., 2002). However, other mRNAs are considered to be efficiently translated during severe vanillin stress, except for *ADH7*.

Shen et al. (2014) recently demonstrated that a vanillin tolerant yeast strain (EMV-8) strongly expressed the *BDH2* gene, which also encodes a putative medium-chain alcohol dehydrogenase/reductase (MDR; Nordling et al., 2002), suggesting the importance of Bdh2 in vanillin tolerance in yeast cells. The amino acid sequence of Bdh2 is 51% identical to that of Bdh1 (González et al., 2010). Although Bdh1 exhibits butanediol-dehydrogenase activity (González et al., 2010), Bdh2 is devoid of this activity and its physiological function remains unclear. The *BDH1* (*YAL060w*) gene is adjacent to the *BDH2* (*YAL061w*) gene in the same chromosome and their transcription is reciprocally regulated (González et al., 2010).

In order to evaluate the *in vivo* roles of Bdh1/Bdh2 in response to vanillin stress, we examined the expression of the *BDH1* and *BDH2* genes under severe vanillin stress conditions in addition to their contribution to the detoxification of vanillin. *bdh2*Δ cells grew slowly in the presence of vanillin with a slower rate of vanillin reduction than wild-type and *bdh1*Δ cells, suggesting that Bdh2 significantly contributes to the detoxification of vanillin *in vivo*. We found that although *BDH2* mRNA was efficiently translated, *BDH1* and *BDH2* mRNA levels were both up-regulated in the presence of severe vanillin stress. We also showed that the *BDH2* promoter enabled the protein synthesis of non-native genes such as *ADH6* and *GFP* under severe vanillin stress, indicating that the *BDH2* promoter is useful for improving the stress tolerance and fermentation efficiency of yeast cells by modifying gene expression in lignocellulose hydrolysates.

## Materials and Methods

### Strains and Medium

*Saccharomyces cerevisiae* strain BY4742 (*MAT*α *his3*Δ*1 ura3*Δ*0 leu2*Δ*0 lys2*Δ*0*) and its isogenic *bdh2*Δ and *bdh1*Δ null mutants (Open Biosystems Inc., Huntsville, AL, USA) were used in the present study. Since the *BDH1* (*YAL060w*) gene is adjacent to the *BDH2* (*YAL061w*) gene in the same chromosome, the double knockout mutant *bdh1*Δ*bdh2*Δ was constructed using the sequential integrative transformation method (Kitada et al., 1995; González et al., 2010). The DNA fragment (1.8 kb) encoding the *bdh1*Δ*bdh2*Δ*::cgHIS3* region was amplified using the plasmid pSHB1805 (Kitada et al., 1995) as a template and the primer set *bdhKO*-F/R (**Table 1**), and was introduced into BY4742. The disruption of the *BDH1* and *BDH2* genes was confirmed by PCR. Cells were cultured in 50 ml of SD medium (2% glucose, 0.67% yeast nitrogen base w/o amino acids, 20 mg/L uracil, 30 mg/L L-lysine HCl, 100 mg/L L-leucine, and 20 mg/L L-histidine HCl) at 28°C with reciprocal shaking (120 rpm) in Erlenmeyer flasks (200 ml). Cell growth in the presence of vanillin was monitored by measuring optical density at 600 nm (OD_600_).

**Table 1:** List of primers used in knockout-mutant and plasmid construction.

Name	Sequence
*bdhKO*-F	5′-TAAATTCATTGAACATATTTCAGAATGAGAGCCTT
	AGCGTATTTCGGTGTTGTAAAACGACGGCCAGT-3′
*bdhKO*-R	5′-AGGACCAACCTTGGAAACAATTCCTGACATCTCAT
	GGCCCATTGCCAGCACAGGAAACAGCTATGACC-3′
*BDH1*-F1	5′-TGAAGGTTGGCGGCCGCGTGGTCGTTGATG-3′
*BDH1*-R1	5′-CCCCAAATATTATTTTCTCGAGACTTCATTTC
	ACCGTG-3′
*BDH1*-F2	5′-GCCGCCTCGAGCTGACTACAAGGATGACGATGAC
	AAGTAATGACAAAATAATATTTGGGGCC-3′
*BDH1*-R2	5′-TCTTTGGATCCAGCAATGCCTCATTCTTGG-3′
*BDH1*-F3	5′-GTACACAGGATCCATGTGCTACACACACCA-3′
*BDH1*-R3	5′-GAGTTGGCCAGTTTGTCTTCTCGAGCCTTT-3′
*BDH1*-R4	5′-TCTTTGGTACCAGCAATGCCTCATTCTTGG-3′
*BDH2*-F1	5′-GATGAACTAGTCTCACTGCTACAAAGTACC-3′
*BDH2*-R1	5′-CAATCCTCGAGATGTGTGACGCAGTTTAGC-3′
*BDH2*-F2	5′-GCAAGCTCGAGCTGACTACAAGGATGACGATGA
	CAAGTGATTGTGATTGAGTACTCACGTTC-3′
*BDH2*-R2	5′-GGCTAGGTACCCGTAAGGAGGAAAGAATAG-3′
*BDH2*-F3	5′-ACTGCCGAGCTCGTCGATCAAGAACTAAAG-3′
*BDH2*-R3	5′-ACGCTAAGGCTCTCATCTAGAAATATGTTC-3′
*BDH2*-F4	5′-GAATTCTCGAGAAGTAACTTGGACTGCCGA-3′
*BDH2*-R4	5′-ACGAAGGATCCGCCCCACTTTTATATGTCG-3′
*GFP*-F	5′-CCAACGAATTCCCGAGCTATGGCTAGCAAA-3′
*GFP*-R	5′-TTCCTCTCGAGCTTTGTTAGCAGCCGGATC-3′

### Plasmids

The oligonucleotide sequences of the primers used for plasmid construction are listed in **Table 1**. Genomic DNA from BY4742 was used as a template to amplify yeast genes by PCR. YIp-*ADH7-FLAG* and pRS316-*ADH7*_Pro_-*ADH6*-*FLAG*-*ADH7*_Ter_ were described previously (Nguyen et al., 2015).

#### YIp-*BDH1*-*GFP* and YIp*-BDH1-FLAG*

A 0.9-kbp fragment encoding part of the open reading frame (ORF) of the *BDH1* gene was amplified using the primer set *BDH1*-F1/R1. The amplicon was digested with *Not*I/*Xho*I, and then cloned into the *Not*I/*Xho*I sites of pJK67 (Kahana et al., 1998) in order to construct YIp-*BDH1*-*GFP*. The plasmid was linearized by *Sph*I and integrated at the chromosomal *BDH1* locus.

The integrate-type plasmid YIp-*BDH1*-*FLAG* was constructed to estimate the protein levels of Bdh1. This plasmid contained a FLAG tag sequence (encoded by 24 nt) immediately upstream of the stop codon and 3′-flanking region of *BDH1*. A 0.4-kbp fragment encoding a FLAG tag sequence, stop codon, and the 3′-flanking region of *BDH1* was amplified using the primer set *BDH1*-F2/R2. The amplicon was digested with *Xho*I/*Bam*HI and cloned into the *Xho*I/*Bam*HI sites of YIp-*BDH1*-*GFP* to construct YIp-*BDH1*-*FLAG*. In order to integrate the *BDH1*-*FLAG* gene at the chromosomal *BDH1* locus, YIp-*BDH1-FLAG* was linearized through its digestion with *Sph*I and was then introduced into yeast cells.

#### YIp-*BDH2*-*GFP* and YIp*-BDH2-FLAG*

A 0.8-kbp fragment encoding part of the ORF of the *BDH2* gene was amplified using the primer set *BDH2*-F1/R1. The amplicon was digested with *Spe*I/*Xho*I, and then cloned into the *Xba*I/*Xho*I sites of pJK67 to construct YIp-*BDH2-GFP*. The plasmid was linearized by *Nar*I and integrated at the chromosomal *BDH2* locus.

The integrate-type plasmid YIp-*BDH2*-*FLAG* was constructed to estimate the protein levels of Bdh2. A 0.35-kbp fragment encoding a FLAG tag sequence, stop codon, and the 3′-flanking region of *BDH2* was amplified using the primer set *BDH2*-F2/R2. The amplicon was digested with *Xho*I/*Kpn*I and cloned into the *Xho*I/*Kpn*I sites of YIp-*BDH2*-*GFP* to construct YIp-*BDH2*-*FLAG*. In order to integrate the *BDH2*-*FLAG* gene at the chromosomal *BDH2* locus, YIp-*BDH2-FLAG* was linearized by digesting with *Nar*I and was then introduced into yeast cells.

#### pRS423-*BDH1* and pRS426-*BDH2*

A DNA fragment containing the promoter region (0.7 kbp), ORF, and terminator region (0.5 kbp) of the *BDH1* gene was amplified using the primer set *BDH1*-F3/R3 and cloned into the *Bam*HI/*Xho*I sites of pRS423 (Sikorski and Hieter, 1989) to construct pRS423-*BDH1*. A DNA fragment containing the promoter region (0.7 kbp), ORF, and terminator region (0.5 kbp) of the *BDH2* gene was amplified using the primer set *BDH2*-F4/R4 and cloned into the *Bam*HI/*Xho*I sites of pRS426 (Sikorski and Hieter, 1989) to construct pRS426-*BDH2*.

#### pRS316-*BDH2*_Pro/FLAG/Ter_

The promoter region (0.7 kbp) and terminator region with the FLAG tag (0.35 kbp) of the *BDH2* gene were amplified using the primer sets *BDH2*-F3/R3 and *BDH2*-F2/R2, respectively. They were cloned into the *Sac*I/*Xba*I and *Xho*I/*Kpn*I sites of pRS316 (Sikorski and Hieter, 1989) to construct pRS316-*BDH2*_Pro/FLAG/Ter_. The ORF of the *GFP* gene was amplified using the primer set *GFP*-F/R, and cloned into the *Xba*I/*Xho*I sites of pRS316-*BDH2*_Pro/FLAG/Ter_ to construct pRS316-*BDH2*_Pro_-*GFP*-*FLAG-BDH2*_Ter_. In order to construct pRS316-*BDH2*_Pro_-*ADH6-FLAG-BDH2*_Ter_, the ORF of the *ADH6* gene was amplified using the primer set *ADH6*_Orf_-F2/R (Nguyen et al., 2015), and cloned into the *Eco*RI/*Xho*I sites of pRS316-*BDH2*_Pro/FLAG/Ter_.

#### pRS316-*BDH2*_Pro_*-ADH6*-*FLAG*-*ADH6*_Ter_

A 0.5-kbp fragment encoding a FLAG tag sequence, stop codon, and the 3′-flanking region of *ADH6* was amplified using the primer set *ADH6*_Ter_-F/R (Nguyen et al., 2015). The amplicon was digested with *Xho*I/*Kpn*I and cloned into the *Xho*I/*Kpn*I sites of pRS316-*BDH2*_Pro_-*ADH6*-*FLAG*-*BDH2*_Ter_ to construct pRS316-*BDH2*_Pro_-*ADH6*-*FLAG*-*ADH6*_Ter_.

#### pRS316-*BDH2_Pro_-ADH6-FLAG-BDH1_Ter_*

A 0.4-kbp fragment encoding a FLAG tag sequence, stop codon, and the 3′-flanking region of *BDH1* was amplified using the primer set *BDH1*-F2/R4. The amplicon was digested with *Xho*I/*Kpn*I and cloned into the *Xho*I/*Kpn*I sites of pRS316-*BDH2*_Pro_-*ADH6*-*FLAG*-*BDH2*_Ter_ to construct pRS316-*BDH2*_Pro_-*ADH6*-*FLAG*-*BDH1*_Ter_.

### Quantitative Real-time PCR

The relative mRNA levels of the *BDH1* and *BDH2* genes were measured by quantitative real time-PCR (qRT-PCR). Total RNA was extracted by the method of Schmitt et al. (1990). cDNA was synthesized using ReverTra Ace qPCR RT Master Mix FSQ-201 (Toyobo, Osaka, Japan), and an analysis was performed using SYBR^®^ Premix Ex Taq^TM^II (Takara Bio Inc., Shiga, Japan) and the sequence detection system (Thermal Cycler Dice Real Time System Lite, Takara Bio Inc., Shiga, Japan). The oligonucleotide sequences of the primers used for qRT-PCR were verified in previous studies (Subramanian et al., 2005; Erasmus and van Vuuren, 2009; Takahashi et al., 2011) and are listed in **Table 2**.

**Table 2:** List of primers used in quantitative real time-PCR.

Gene	Orientation	Primer sequence
*BDH1*	Forward	5′-TGACTGGTTCGATCGGCTATG-3′
	Reverse	5′-TGGATGGCACGAACAACTTC-3′
*BDH2*	Forward	5′-GAGAGCCTTAGCGTATTTCG-3′
	Reverse	5′-CCTGTGGTATCTGCGGATCT-3′
*ACT1*	Forward	5′-TTGGATTCCGGTGATGGTGTTACT-3′
	Reverse	5′-TGAAGAAGATTGAGCAGCGGTTTG-3′

### Chemicals and Analysis Methods

Vanillin, furfural, and dimethyl sulfoxide (DMSO) were obtained from Wako (Osaka, Japan). Cycloheximide (CHX) was purchased from Nacalai Tesque, Kyoto, Japan. Stock solutions of 2 M vanillin, 2 M furfural, and 20 mg/ml CHX were prepared in DMSO and stored at -30°C. Vanillin levels in SD medium were assayed by the methods of Fitzgerald et al. (2003). Briefly, after spinning down cells, the supernatant was separated using a linear gradient elution of 10–45% acetonitrile in 0.1% trifluoroacetic acid by high performance liquid chromatography (HPLC; Shimadzu, Kyoto, Japan) equipped with a C18 reversed-phase column, TSKgel ODS-100S (4.6 mm × 150 mm; Tosoh, Tokyo, Japan). The column was maintained at 40°C. The eluted compounds were detected at 280 nm. A Leica AF6500 (Leica Microsystems Vertrieb GmbH, Germany) fluorescence microscopic system was used for the microscopic analysis. Cells treated with vanillin were immediately observed without fixation. All experiments were repeated at least three times.

### Western Blot Analysis

A cell-free extract (CFE) in 50 mM potassium phosphate buffer (pH 6.8) was prepared after the stress treatment. The total protein concentrations of CFE were measured using the Protein Assay CBB Solution kit (Nacalai Tesque, Kyoto, Japan) and 36 micrograms of total protein was applied to each lane of a 12.5% polyacrylamide gel for the SDS-PAGE analysis. The levels of FLAG-tagged proteins (Bdh1, Bdh2, Adh6, and Adh7) were monitored using a monoclonal anti-FLAG M2 antibody (Sigma–Aldrich, St. Louis, MO, USA). GFP levels were monitored with an anti-GFP antibody (mFX75; Wako, Osaka, Japan). Pgk1 was used as a loading control, and its levels were monitored with a monoclonal anti-PGK antibody (22C5D8; Life Technologies, Frederick, MD, USA). The bands of the Western blot were quantified using Image Studio Digits Ver 4.0 software (LI-COR Biotechnology, Lincoln, NE, USA).

## Results

### *bdh2*Δ Mutants Were Sensitive to Vanillin

**FIGURE 1:**
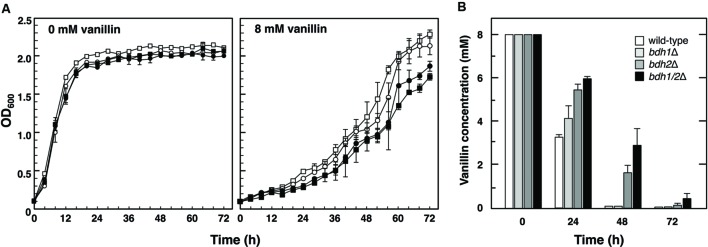
**Growth of *bdh1*Δ, *bdh2*Δ, and *bdh1*Δ*bdh2*Δ mutants in the presence of vanillin. (A)** Cells were cultured at 28°C in SD medium containing 8 mM vanillin. Growth was monitored by measuring the optical density at 600 nm (OD_600_). Data are shown as the mean ± SE of three independent experiments. The strains examined were as follows: *open squares*, wild-type; *open circles*, *bdh1*Δ; *closed circles*, *bdh2*Δ; *closed squares*, *bdh1*Δ*bdh2*Δ. **(B)** Changes in vanillin concentrations in culture media. The concentration of vanillin was determined by high performance liquid chromatography (HPLC). Yeast cells were cultured in SD medium containing 8 mM vanillin, and supernatants of the culture media were collected. Data are shown as the mean ± SE of three independent experiments.

In order to investigate the importance of Bdh1 and Bdh2 for tolerance to vanillin, the growth of the null mutants of *BDH* genes in SD medium containing vanillin (the initial vanillin concentration was 8 mM) was investigated (**Figure 1A**). Although the growth curves of wild-type and the mutants (*bdh1*Δ, *bdh2*Δ, and *bdh1*Δ*bdh2*Δ) were almost identical under non-stressed conditions, the presence of vanillin led to slower growth rates for the *bdh2*Δ and *bdh1*Δ*bdh2*Δ mutants than those for the wild-type and *bdh1*Δ mutant. This result strongly indicates the importance of Bdh2 for vanillin tolerance.

In an attempt to further confirm the importance of Bdh1 and Bdh2, we examined changes in vanillin concentrations in medium throughout the cultivation (**Figure 1B**). After a 24-h cultivation, all mutants were less efficient at reducing vanillin levels than wild-type cells, indicating that Bdh1 and Bdh2 contribute to reductions in vanillin. While almost no vanillin was detected in the media of wild-type and *bdh1*Δ cells after a 48-h cultivation, approximately 2–3 mM vanillin remained in the media of *bdh2*Δ and *bdh1*Δ*bdh2*Δ cells. These results indicate that Bdh2 more effectively contributes to reductions in vanillin levels than Bdh1.

### Synthesis of the Bdh2 Protein Was Inducible with Severe Vanillin Stress

**FIGURE 2:**
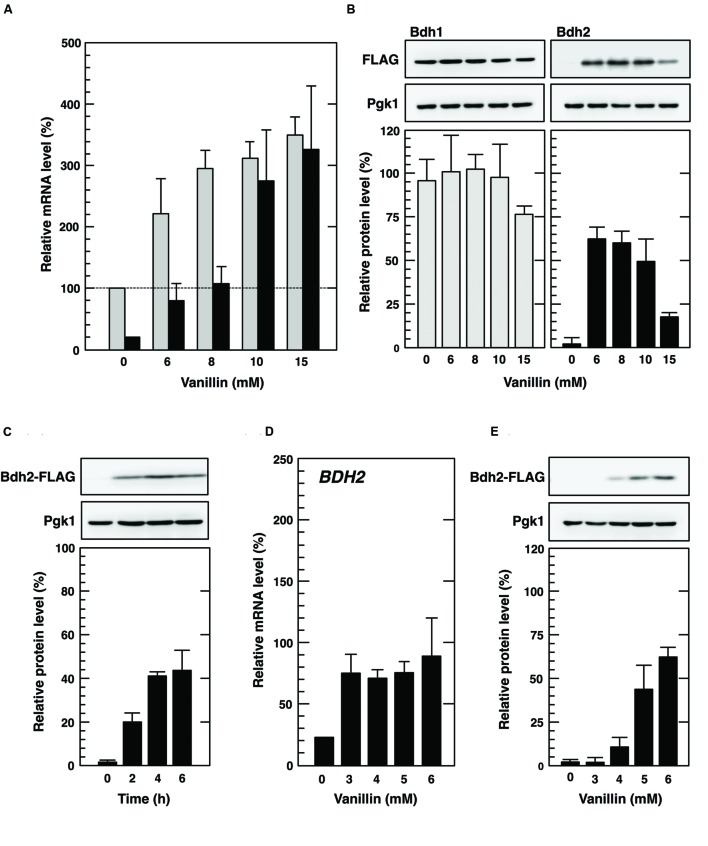
**Expression of *BDH1* and *BDH2* genes under vanillin stress. (A,D)** Cells in the exponential phase of growth were treated with vanillin for 1 h. *BDH1* and *BDH2* mRNA levels were analyzed by qRT-PCR. In order to compare mRNA expression levels, the mRNA level of each gene was normalized to that of *ACT1.* The *BDH1* mRNA level in cells not treated with vanillin was considered to be 100%. Data are shown as the mean ± SE of three independent experiments. *Gray bars*, *BDH1* mRNA; *black bars*, *BDH2* mRNA. **(B,C,E)** Cells carrying a FLAG-tagged chromosomal copy of the *BDH1* or *BDH2* gene in the exponential phase of growth were treated with 6–15 mM vanillin for 1 h **(B)**, 15 mM vanillin for 2–6 h **(C)**, or 3–6 mM vanillin for 1 h **(E)**. Bdh1-FLAG and Bdh2-FLAG levels were determined by Western blot analysis using an anti-FLAG antibody. Pgk1 was used as a loading control. The protein levels of Bdh1-FLAG and Bdh2-FLAG were normalized to that of Pgk1, and the intensity of the Pgk1 band of each lane was considered to be 100%. Data are shown as the mean ± SE of three independent experiments.

Since Bdh1 and Bdh2 both appear to be involved in reductions in vanillin and vanillin tolerance, we next examined whether vanillin affects the transcription of the *BDH1* and *BDH2* genes using quantitative real time-polymerase chain reaction (qRT-PCR). *BDH1* and *BDH2* mRNA levels were both significantly increased by the treatment with vanillin (6–15 mM; **Figure 2A**), indicating that both *BDH* genes were transcriptionally up-regulated by vanillin stress. Notably, the induction of the transcription of *BDH2* by vanillin was markedly greater than that of *BDH1* (**Figure 2A**).

We reported that severe vanillin stress (greater than 7.5 mM) causes a rapid reduction in overall protein synthesis (Nguyen et al., 2014), and a previous study also verified the translational repression caused by vanillin (Nguyen et al., 2015). Therefore, we investigated the effects of severe vanillin stress on the protein synthesis of Bdh1 and Bdh2. Protein levels of Bdh1 were constitutive in the presence of varying concentrations of vanillin. In contrast, the Bdh2 protein was synthesized at levels lower than the detection limit under non-stressed conditions, whereas clear bands representing Bdh2 protein were detected after the treatments with vanillin (**Figure 2B**). Protein synthesis of Bdh2 was still detected following the treatment with 15 mM vanillin, which severely repressed bulk translation activity (Nguyen et al., 2014, 2015). Additionally, a prolonged treatment with 15 mM vanillin elevated Bdh2 protein levels (**Figure 2C**). These results clearly suggest that *BDH2* mRNA can be translated even in the presence of high concentrations of vanillin, despite the repression of overall protein synthesis. On the other hand, Bdh2 protein synthesis was hardly up-regulated in the presence of low concentrations of vanillin (less than 4 mM) despite the increased mRNA levels of *BDH2* (**Figures 2D,E**), clearly suggesting that the expression of the *BDH2* gene was mainly inducible with severe, but not mild vanillin stress.

**FIGURE 3:**
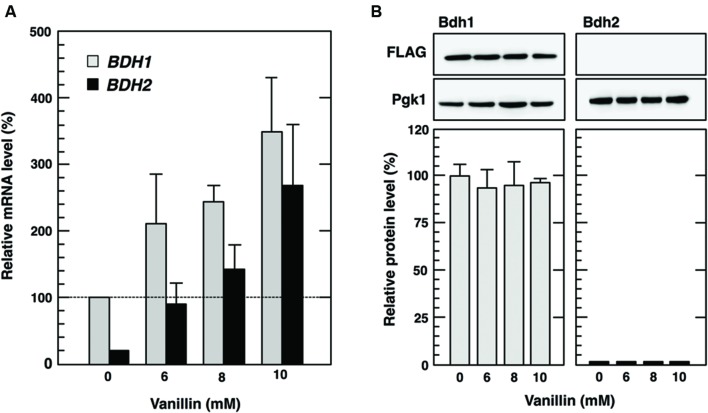
**Cycloheximide had little effect on Bdh1 protein levels under vanillin stress. (A)** Cells in the exponential phase of growth were treated with 6–10 mM vanillin and 0.1 mg/ml cycloheximide (CHX) for 1 h. *BDH1* and *BDH2* mRNA levels were analyzed by qRT-PCR. In order to compare mRNA expression levels, the mRNA level of each gene was normalized to that of *ACT1.* The *BDH1* mRNA level in cells not treated with vanillin was considered to be 100%. Data are shown as the mean ± SE of three independent experiments. *Gray bars*, *BDH1* mRNA; *black bars*, *BDH2* mRNA. **(B)** Cells carrying a FLAG-tagged chromosomal copy of the *BDH1* or *BDH2* gene in the exponential phase of growth were treated with 6–10 mM vanillin and 0.1 mg/ml CHX for 1 h. Bdh1-FLAG and Bdh2-FLAG levels were determined by Western blot analysis using an anti-FLAG antibody. Pgk1 was used as a loading control. The protein levels of Bdh1-FLAG and Bdh2-FLAG were normalized to that of Pgk1, and the intensity of the Pgk1 band of each lane was considered to be 100%. Data are shown as the mean ± SE of three independent experiments.

To verify that translation of *BDH1* mRNA was repressed by vanillin, we also investigated protein levels of Bdh1 in cells treated with vanillin and CHX, a strong inhibitor of protein biosynthesis (Obrig et al., 1971), simultaneously. CHX had almost no effect on the transcriptional activation of *BDH1* and *BDH2* under vanillin stress (**Figures 2A** and **3A**). Since CHX inhibits protein synthesis during the treatment with vanillin, induction of Bdh2 protein synthesis under vanillin stress was almost completely repressed (**Figure 3B**). On the other hand, CHX had almost no effect on the protein levels of Bdh1 after the treatment with vanillin for 1 h (**Figures 2B** and **3B**). Since blocking the supply of newly synthesized protein had little effect on the Bdh1 levels, these results clearly indicate that translation of *BDH1* mRNA was repressed under vanillin stress and also suggest that Bdh1 was relatively stable and its protein turnover was not so fast at least under our experimental conditions.

### Elimination of Vanillin Did Not Maintain the Synthesis of the Bdh2 Protein

**FIGURE 4:**
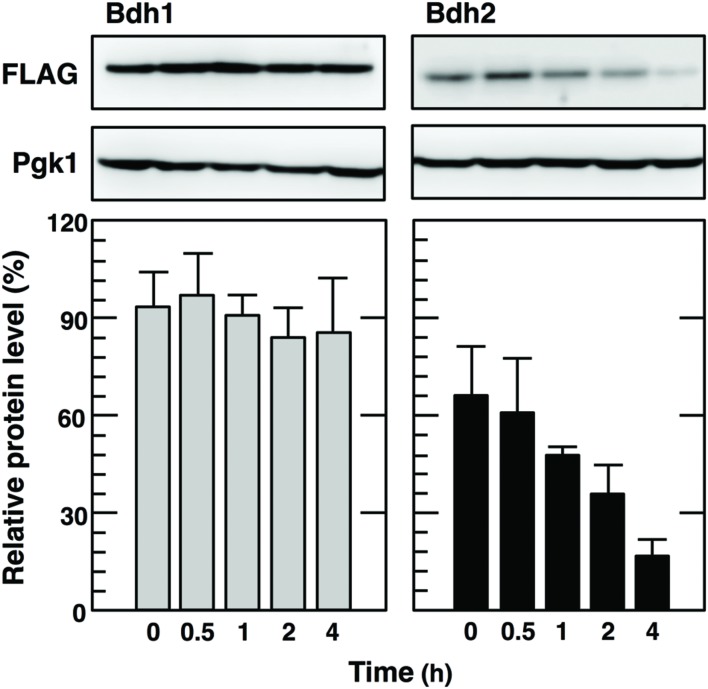
**Bdh2 protein levels gradually decreased with the elimination of vanillin stress**. Cells carrying a FLAG-tagged chromosomal copy of the *BDH1* or *BDH2* gene were treated with 8 mM vanillin for 1 h, washed with 50 mM potassium phosphate buffer (pH 6.8) twice, and then transferred to fresh SD medium without vanillin. Cells were incubated further for the indicated periods of time. Bdh1-FLAG and Bdh2-FLAG levels were determined by Western blot analysis using anti-FLAG antibody. Pgk1 was used as a loading control. The protein level of Bdh2-FLAG was normalized to that of Pgk1, and the intensity of the Pgk1 band in each lane was considered to be 100%. Data are shown as the mean ± SE of three independent experiments.

We next examined the effects of the elimination of vanillin on the increases in Bdh2 protein levels caused by severe vanillin stress. After the treatment with 8 mM vanillin for 1 h, cells were transferred to fresh medium without vanillin. Changes in Bdh1 and Bdh2 protein levels were monitored during the recovery process (**Figure 4**). Although Bdh1 protein levels remained almost constant after the elimination of vanillin, those of Bdh2 were not maintained and gradually decreased during the recovery process and were finally lower than the detection limit after 5 h. These results suggest that the expression of *BDH2* is repressed under non-severe vanillin stress conditions.

### Overexpression of the *BDH2* Gene Improved Yeast Vanillin Tolerance

**FIGURE 5:**
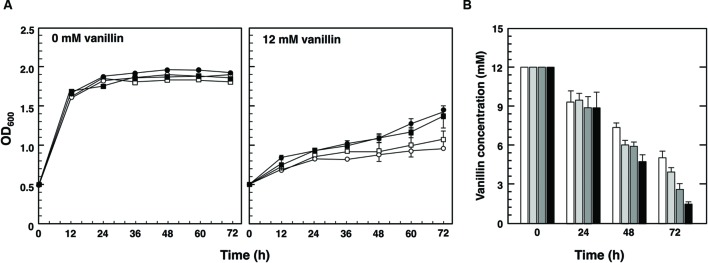
**A high-copy plasmid containing the *BDH2* gene improved vanillin tolerance in yeast cells**. Cells were cultivated in SD medium containing 0 or 12 mM vanillin at 28°C. The plasmids introduced into cells were as follows: *open squares* and *white bars*, pRS423 and pRS426; *open circles* and *light gray bars*, pRS423-*BDH1* and pRS426; *closed circles* and *dark gray bars*, pRS423 and pRS426-*BDH2*; *closed squares* and *black bars*, pRS423-*BDH1* and pRS426-*BDH2*. **(A)** Growth was monitored by measuring optical density at 600 nm (OD_600_). Data are shown as the mean ± SE of three independent experiments. **(B)** Vanillin in SD medium was monitored by HPLC. Data are shown as the mean ± SE of three independent experiments.

We also investigated the effects of the overexpression of the *BDH2* gene on tolerance to severe vanillin stress. Cells carrying the *BDH2*-overexpression plasmid (pRS426-*BDH2*) showed better growth in medium with very severe vanillin stress (the initial concentration of 12 mM) than cells carrying the control plasmid (pRS426; **Figure 5A**). On the other hand, the growth of cells carrying pRS423-*BDH1* and pRS426 was slightly better than that of cells carrying empty plasmids only (pRS423 and pRS426) in the presence of 12 mM vanillin, indicating that the overexpression of the *BDH1* gene only has a little effect on tolerance to severe vanillin stress. Additionally, vanillin levels in the culture medium were determined by HPLC (**Figure 5B**). The overexpression of *BDH2* efficiently reduced of vanillin levels in the medium. The overexpression of *BDH1* also facilitated reductions in vanillin levels; however, its effects were less than those of *BDH2.* These results strongly indicate that Bdh2 plays a very important role in vanillin detoxification and tolerance.

### The *BDH2* Promoter Enabled Protein Synthesis under Severe Vanillin Stress

**FIGURE 6:**
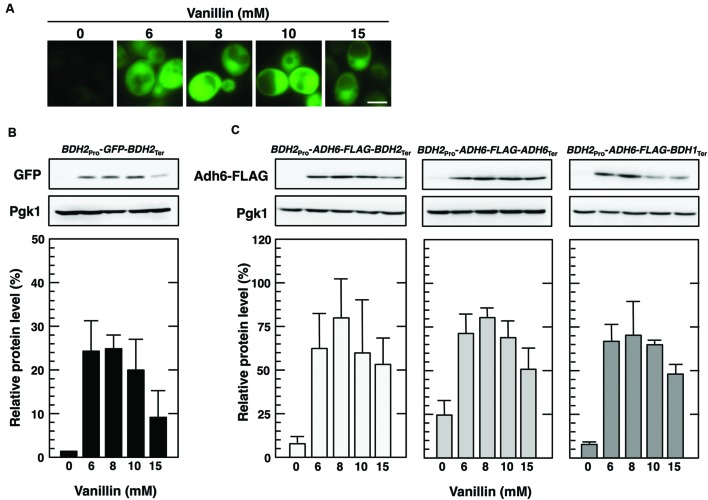
**The *BDH2* promoter region enabled protein synthesis of non-native genes in the presence of high concentrations of vanillin. (A,B)** Cells carrying pRS316-*BDH2*_Pro_-*GFP-FLAG-BDH2*_Ter_ in the exponential phase of growth were treated with vanillin for 1 h. **(A)** A fluorescence microscopic analysis was performed to monitor the synthesis of GFP. The exposure times were the same for all conditions. White bar indicates 3 μm. **(B)** GFP protein levels were determined by Western blot analysis using an anti-GFP antibody. **(C)** Cells carrying pRS316-*BDH2*_Pro_-*ADH6-FLAG-BDH2*_Ter_, pRS316-*BDH2*_Pro_-*ADH6-FLAG-ADH6*_Ter_, or pRS316-*BDH2*_Pro_-*ADH6-FLAG-BDH1*_Ter_ in the exponential phase of growth were treated with vanillin for 1 h. Protein levels of Adh6-FLAG were determined by Western blot analysis using an anti-FLAG antibody. Pgk1 was used as a loading control. Protein levels of GFP and Adh6-FLAG were normalized to those of Pgk1, and the intensity of the Pgk1 band in each lane was considered to be 100%. Data are shown as the mean ± SE of three independent experiments.

Previous studies reported that the promoters of small heat shock protein genes are sufficient for efficient protein synthesis upon glucose depletion, which causes the repression of bulk translation activity (Ashe et al., 2000; Zid and O’Shea, 2014). We also recently demonstrated that the *ADH7* promoter enables effective protein synthesis, even under severe vanillin stress (Nguyen et al., 2015). Furthermore, we examined whether the *BDH2* promoter enables protein synthesis under severe vanillin stress. The *BDH2* promoter and terminator regions were fused to other ORFs such as *GFP* and *ADH6*. Cells carrying pRS316-*BDH2*_Pro_-*GFP*-*BDH2*_Ter_ showed a clear GFP signal after the treatment with vanillin, however, the signal was hardly detected under non-stressed conditions (**Figure 6A**). Increases in the protein levels of GFP were verified by Western blot analysis (**Figure 6B**). Furthermore, Adh6-FLAG protein levels were increased by severe vanillin stress in cells carrying pRS316-*BDH2*_Pro_-*ADH6-FLAG-BDH2*_Ter_ (**Figure 6C**). These results strongly indicate that the *BDH2* promoter region enables the expression of its regulated genes under severe vanillin stress. Since the increased protein levels of Adh6-FLAG were also caused by severe vanillin stress in cells carrying pRS316-*BDH2*_Pro_-*ADH6*-*FLAG*-*ADH6*_Ter_ or pRS316-*BDH2*_Pro_-*ADH6-FLAG-BDH1*_Ter_ (**Figure 6C**), the *BDH2* terminator region appears to have a negligible effect on preferential translation upon severe vanillin stress.

### Furfural also Induced Protein Synthesis under the Control of the *BDH2* Promoter

**FIGURE 7:**
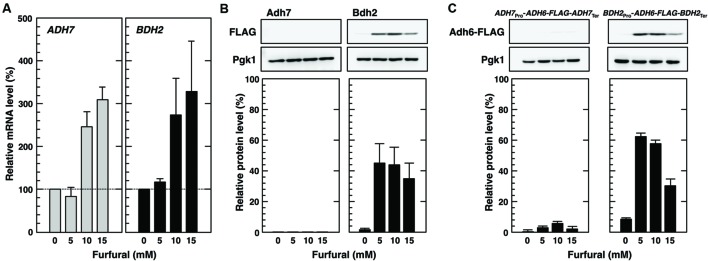
**Furfural induced the expression of *BDH2* promoter-driven genes. (A)** Cells in the exponential phase of growth were treated with furfural (5–15 mM) for 3 h. *ADH7* and *BDH2* mRNA levels were analyzed by qRT-PCR. In order to compare mRNA expression levels, the mRNA level of each gene was normalized to that of *ACT1.* The mRNA level in cells not treated with furfural was considered to be 100%. Data are shown as the mean ± SE of three independent experiments. **(B,C)** Cells carrying a FLAG-tagged chromosomal copy of the *ADH7* or *BDH2* gene **(B)**, and cells carrying pRS316-*BDH2*_Pro_-*ADH6-FLAG-BDH2*_Ter_ or pRS316-*ADH7*_Pro_-*ADH6-FLAG-ADH7*_Ter_ **(C)** in the exponential phase of growth were treated with furfural (5–15 mM) for 3 h. Adh7-FLAG, Bdh2-FLAG, and Adh6-FLAG protein levels were determined by Western blot analysis using an anti-FLAG antibody. Pgk1 was used as a loading control. FLAG-tagged protein levels were normalized to those of Pgk1, and the intensity of the Pgk1 band of each lane was considered to be 100%. Data are shown as the mean ± SE of three independent experiments.

During the process of bioethanol production from the lignocellulosic biomass, yeast cells are exposed to various inhibitory compounds in lignocellulose hydrolysates. Furfural is one of the major biomass conversion inhibitors from the lignocellulosic biomass and affects yeast fermentation activity (Klinke et al., 2004; Lu et al., 2009). Additionally, high concentrations of furfural have been shown to repress translation activity (Iwaki et al., 2013a). Therefore, we examined whether the protein synthesis of Bdh2 was induced under furfural stress conditions. After a treatment with 5–15 mM furfural for 3 h, *BDH2* mRNA levels and the protein synthesis of Bdh2 were increased (**Figures 7A,B**). Furthermore, we investigated whether the *BDH2* promoter enabled protein synthesis under furfural stress conditions. Cells carrying pRS316-*BDH2*_Pro_-*ADH6-FLAG-BDH2*_Ter_ showed increased protein levels of Adh6-FLAG in the presence of furfural (**Figure 7C**). These results indicate that the *BDH2* promoter is useful for efficient gene expression under furfural stress conditions as well as severe vanillin stress. On the other hand, the protein synthesis of Adh7 and *ADH7* promoter-driven Adh6-FLAG (Nguyen et al., 2015) was not significantly induced by furfural (**Figure 7**).

## Discussion

We herein demonstrated the importance of *BDH1* and *BDH2* genes in tolerance to vanillin stress even though they were not detected as genes involved in vanillin tolerance in previous genome-wide studies (Endo et al., 2008; Pereira et al., 2011). The null mutants of *BDH* genes were more sensitive to vanillin than the wild-type. *bdh2*Δ cells showed hyper-sensitivity to vanillin and reduced vanillin less efficiently than wild-type cells and *bdh1*Δ cells (**Figure 1**), thereby verifying the significant contribution of Bdh2 to vanillin tolerance. Therefore, cells carrying the *BDH2*-overexpression plasmid (pRS426-*BDH2*) showed a significant improvement in growth under very severe vanillin stress (12 mM; **Figure 5**). Bdh1 and Bdh2 as well as Adh6 and Adh7 belong to the superfamily of MDR (Ehsani et al., 2009). Since Adh6 and Adh7 catalyze the reduction of vanillin to vanillyl alcohol (Larroy et al., 2002), it appears to be quite feasible that Bdh1 and Bdh2 also catalyze the reduction of vanillin to vanillyl alcohol.

We demonstrated that the *BDH1* and *BDH2* genes exhibited different protein expression patterns in response to vanillin stress. Bdh1 protein levels were almost constitutive and did not reflect the increased levels of *BDH1* mRNA upon severe vanillin stress (**Figure 2**). Since blocking protein synthesis using CHX had almost no effect on the Bdh1 protein levels (**Figure 3**), it is likely that translation of *BDH1* mRNA was repressed under severe vanillin stress. On the other hand, *BDH2* mRNA could be translated upon severe vanillin stress, and Bdh2 levels were increased even in the presence of a very high concentration of vanillin (15 mM; **Figures 2B,C**). These results revealed that the expression of the *BDH2* gene was inducible upon severe vanillin stress. The synthesis of the Bdh2 protein was not induced by low concentrations of vanillin (1–4 mM) in spite of the transcriptional activation of *BDH2* (**Figures 2D,E**), suggesting that the expression of *BDH2* under 1–4 mM vanillin stress is repressed in a posttranscriptional manner, which still remains to be clarified. Since Bdh2 levels were gradually reduced after the elimination of vanillin stress (**Figure 4**), protein degradation may also be important to maintain the low levels of Bdh2 under non-stress and mild vanillin stress conditions.

We recently reported that the synthesis of the Adh7 protein but not the Adh6 protein was induced by severe vanillin stress, despite both *ADH* genes being transcriptionally up-regulated (Nguyen et al., 2015). Additionally, the expression of *ADH7* was not induced by mild vanillin stress (1–4 mM; Nguyen et al., 2015). Therefore, it is clear that the expression pattern of *BDH2* closely resembles that of *ADH7* in response to vanillin stress. The induction of *BDH2* and *ADH7* expression by severe vanillin stress appears to be crucial for yeast tolerance to high concentrations of vanillin. On the other hand, the constitutive expression of *BDH1* and *ADH6* may play a role in the initial stage of the response to vanillin, and yeast cells are presumably able to cope with mild vanillin stress without the expression of *BDH2* and *ADH7.* Adh6 and Adh7 are NADP(H)-dependent MDRs, while Bdh1 and Bdh2 are NAD(H)-dependent MDRs (González et al., 2000; Ehsani et al., 2009). The use of both kinds of reductants for vanillin reduction appears to be a strong advantage for maintaining the robustness of the yeast response to vanillin stress.

It has been previously reported that specific promoter sequences affected the efficiency of translation under glucose depletion, which causes severe bulk translation repression (Zid and O’Shea, 2014). They demonstrated that the transcripts of genes containing heat shock elements (HSEs) in their promoter regions may be preferentially translated during glucose starvation. It is also conceivable that *BDH2* and *ADH7* also have specific promoter sequences that enable preferential translation under severe vanillin stress. HSEs (5′-NGAANNTTCN-3′ or 5′-NTTCNNGAAN-3′) have been detected in *BDH2* promoter sequences (from positions -389 to -378 and -119 to -108, relative to the translation initiation ATG codon) and *ADH7* promoter sequences (-473 to -464; Bonner et al., 1994). On the other hand, no HSE has been detected in the promoter regions of *BDH1* and *ADH6.* HSEs in the promoters of *BDH2* and *ADH7* may also contribute to preferential translation under severe vanillin stress as well as glucose depletion conditions. In mammalian cells, the recruitment of the translation elongation factor eEF1A to the HSEs of the *HSP70* gene was found to promote the nuclear export and translation of *HSP70* mRNA during heat shock (Vera et al., 2014). Similar to mammalian eEF1A, some kinds of factor(s) that facilitate translation under severe stress conditions may be recruited to HSEs in yeast cells.

In addition to the *ADH7* promoter, the ability of the *BDH2* promoter to overcome vanillin-induced translation repression appears to be useful for improving the efficiency of bioethanol production. Since the expression of *BDH2* but not *ADH7* was also induced by furfural (**Figure 7**), the *BDH2* promoter may be more practical than the *ADH7* promoter for efficient bioethanol production. The manipulation of gene expression using the *BDH2* promoter may represent an effective approach in the breeding of robust and optimized yeast strains for bioethanol production. For example, the expression of UDP-glucoside transferase using a *BDH2* promoter may efficiently reduce vanillin levels through the conversion of vanillin to vanillin β-D-glucoside (Hansen et al., 2009; Brochado et al., 2010; Brochado and Patil, 2013; Gallage and Møller, 2015), and vanillin β-D-glucoside may be collected and re-converted into vanillin after the completion of fermentation. Although further studies are needed in order to construct an ideal high-performance yeast for bioethanol production from the lignocellulosic biomass, the results of the present study provide useful information on yeast responses to vanillin and insights in order to improve the vanillin tolerance and fermentation efficiency of yeast cells.

## Author Contributions

YI did most of experiments and prepared the manuscript. TN and SK did several important experiments. SI did several experiments and mainly prepared the manuscript.

## Conflict of Interest Statement

The authors declare that the research was conducted in the absence of any commercial or financial relationships that could be construed as a potential conflict of interest.
